# Optimizing Phycocyanin Extraction from Cyanobacterial Biomass: A Comparative Study of Freeze–Thaw Cycling with Various Solvents

**DOI:** 10.3390/md22060246

**Published:** 2024-05-28

**Authors:** Konstantinos Pispas, Georgios Manthos, Eirini Sventzouri, Maria Geroulia, Savvas Giannis Mastropetros, Sameh Samir Ali, Michael Kornaros

**Affiliations:** 1 Laboratory of Biochemical Engineering & Environmental Technology (LBEET), Department of Chemical Engineering, University of Patras, 26504 Patras, Greece; pispas.msci@gmail.com (K.P.); geomanthos@chemeng.upatras.gr (G.M.); eirinisventzouri@gmail.com (E.S.); up1069034@upnet.gr (M.G.); savvasgiannismas@gmail.com (S.G.M.); 2 Botany Department, Faculty of Science, Tanta University, Tanta 31527, Egypt; 1000004956@ujs.edu.cn

**Keywords:** pigments, phycocyanin, extraction, freeze–thaw, extraction, cyanobacteria

## Abstract

Cyanobacterial phycocyanin pigment is widely utilized for its properties in various industries, including food, cosmetics, and pharmaceuticals. Despite its potential, challenges exist, such as extraction methods impacting yield, stability, and purity. This study investigates the impact of the number of freeze–thaw (FT) cycles on the extraction of phycocyanin from the wet biomass of four cyanobacteria species (*Arthrospira platensis*, *Chlorogloeopsis fritschii*, *Phormidium* sp., and *Synechocystis* sp.), along with the impact of five extraction solutions (Tris-HCl buffer, phosphate buffer, CaCl_2_, deionized water, and tap water) at various pH values. *Synechocystis* sp. exhibited the highest phycocyanin content among the studied species. For *A. platensis*, Tris-HCl buffer yielded maximum phycocyanin concentration from the first FT cycle, while phosphate buffer provided satisfactory results from the second cycle. Similarly, Tris-HCl buffer showed promising results for *C. fritschii* (68.5% of the maximum from the first cycle), with the highest concentration (~12% *w*/*w*) achieved during the seventh cycle, using phosphate buffer. *Phormidium* sp. yielded the maximum pigment concentration from the first cycle using tap water. Among species-specific optimal extraction solutions, Tris-HCl buffer demonstrated sufficient extraction efficacy for all species, from the first cycle. This study represents an initial step toward establishing a universal extraction method for phycocyanin from diverse cyanobacteria species.

## 1. Introduction

Natural microbial pigments are one of the most researched products in the food industry. Their usage can effectively point out food’s sensorial value and can also be an indicator of its freshness and good condition [1]. Additionally, microbial pigments have been proven as beneficial materials that can promote a healthy way of life, providing nutrients and trace elements to the consumers [2]. Although most of the pigment production strategies are already developed, blue natural pigments are still of interest to researchers and companies in order to improve the sustainability of food production activities [3].

Among the other natural pigments, phycocyanin (PC) exhibits a wide range of applications in different industrial sectors, from food coloring to pharmaceuticals and cosmetics [4,5]. PC is a multi-chain protein composed of homologous sub-chains. The first chain is constructed by one phycocyanobilin bonding at cysteine, and the second by two phycocyanobilins bonding with two different types of cysteine (84 and 155) [6]. Its structure makes it suitable for various applications in drug development since it presents an increased antioxidant activity that can prevent diseases, such as cancer, inflammation, and other human disorders [7]. It is worth noting that PC purity is a crucial factor affecting its application. PC purity greater than 0.7 indicates that the pigment could be used in food products, while cosmetic dye PC application can be achieved when its purity is greater than 2.0. Analytical-grade PC can be considered when its purity is higher than 4.0 [8]. The cyanobacterium *Spirulina platensis* is the most common microorganism for the production of PC on an industrial scale, having the ability to accumulate large quantities of this pigment in its biomass cells [9]. Despite the wide product applicability, there are several challenges regarding the optimum extraction method for maximum protein recovery from the cells while maximizing the product’s purity [10]. Several methods have been proposed in order to maximize phycocyanin purity. Precipitation and redissolving have been proposed by Silva et al. [11], while ultrafiltration has been investigated by Figueira et al. [8]. Greater PC purity can be achieved using ion exchange chromatography combined with physicochemical treatment, increasing the process’s complexity and cost [12]. 

Several different techniques have been proposed for phycocyanin extraction in laboratory and large-scale applications. Most of them include biomass harvesting and drying before the addition of the solvent. Nevertheless, PC extraction from wet biomass exhibited greater yields [13]. Moreover, the solvent-to-biomass ratio has been recognized as a crucial parameter for the yield maximization [14]. Other researchers have proposed alternative physical or chemical methods for phycocyanin recovery. These methods primarily include sonication [15,16], enzymic treatment [17], homogenization [18], pulsed electric field [13,17], acid treatment, bead beating [16], microwaves [19], and combinations of these. Most of these studies focus on a sustainable and economically efficient method for high-added-value product isolation from biomass cells. For instance, in the work of İlter et al. [20], the phycocyanin extraction from wet, frozen, and dry biomass of *Spirulina platensis* was tested under different extraction techniques (classical, ultrasound, and microwave). The frozen biomass exhibited an increased yield value compared to the wet and dried biomass (5 times higher yield than dried biomass, and 2 times higher yield than wet biomass). Additionally, the ultrasound method was obtained as the optimum method. The consecutive freeze and thaw method has been reported as the easiest and most effective technique for PC recovery from cyanobacteria. The phycocyanin extraction yield was exhibited to be highest after the sixth freeze–thaw cycle for certain cyanobacteria (*N. commune* and *O. okeni*) but this is not a universally applicable fact for all the different species [13]. In another study of Khandual et al. [21], high PC yield and purity were exhibited for dry biomass of *Arthrospira platensis* (48.8 mg PC g^−1^ biomass and 0.47 purity) using 20 min freeze–thaw cycles and 24 h of agitation at 120 rpm. However, the freeze–thaw method can be energy-intensive, particularly for extractions with many cycles. 

The scope of the present work was the experimental investigation of phycocyanin extraction using the freeze–thaw technique and different solvents. The general methodological framework was initiated with the cultivation of four different species (*Arthrospira platensis*, *Chlorogloeopsis fritschii*, *Phormidium* sp., and *Synechocystis* sp.) under autotrophic conditions in order to accumulate intercellular phycocyanin. Subsequently, the freeze–thaw technique was applied using five different solvents of different pH values. The investigated extraction conditions were classified according to the PC extraction yield and the PC purity. Although PC extraction has been studied in the literature, there is not currently a general technique for the species tested herein using diversified solvents. To this end, this work offers applied information about the different species’ behavior in the multiple commonly used solvents, offering adequate results for perspective understanding of the underlying process mechanisms. This work can provide the extensive conclusions needed for a decision-maker to achieve maximum product recovery and purity for the tested species. Moreover, it can be a first step in the selection of an environmentally and economically sustainable solvent. 

## 2. Results and Discussion

### 2.1. Yield and Purity of Phycocyanin Extracted from Arthrospira platensis

**Figure 1:**
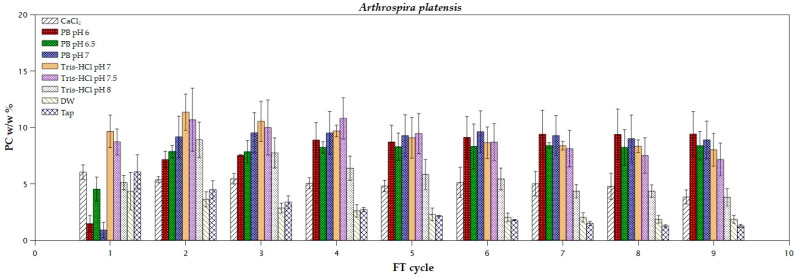
Extraction yields of phycocyanin from *Arthrospira platensis* using different solvents during freeze and thaw cycles.

*A. platensis* biomass exhibited the highest PC yield (11.34% of dry cell weight) after the first cycle of extraction with 1 M Tris-HCl buffer (pH 7; Figure 1). Tris-HCl buffer is used as a lysis buffer due to its effectiveness in extracting both soluble cytoplasmic and transmembrane proteins [22,23]. This makes it a versatile choice for isolating proteins from various cellular compartments, such as the nucleus, chloroplasts, and cell membrane [23]. This yield is higher than those reported by Tavanandi et al. (7.45% *w*/*w*, 4 FT cycles) [18] and Gorgich et al. (4.42% *w*/*w*) [24]. However, Tris-HCl resulted in moderately low phycocyanin purity (<0.4; Table 1), possibly due to the co-extraction of other pigments (β-carotene) from the biomass [25]. This decrease in purity could also be observed visually from the color fading of the supernatant, from vivid blue to green. While phosphate buffer is considered a good choice for phycocyanin extraction from *Arthrospira* species by many researchers [24,26,27], it could not extract a reasonable amount of PC in the first FT cycle compared to Tris-HCl. Notably, phosphate buffer at pH 6.5 achieved a higher PC extraction efficiency during the first FT cycle, compared to the other pH values. Acidic aqueous solutions can increase the solubility of phycocyanin by promoting its protonation [28]. Moreover, pH strongly affects the stability of the extracted PC over time. The optimal pH values range between 6 and 7, while in neutral pH the pigment is less stable [9,26,28]. However, its performance seemed satisfactory in the second cycle (pH 7, 9.16% *w*/*w*, purity 0.71). Calcium chloride (0.1 M), which contributes to the disruption of the cell by changing the osmotic pressure in the solution [29], extracted a sufficient amount of PC during the first cycle and yielded the highest purity (0.91) for *A. platensis*, as already mentioned by Schipper et al. [16]. Unlike phosphate buffer, which can disrupt the cell wall and cause the release of other pigments, calcium chloride dissolves the sodium-calcium canal, allowing PC accumulation within the cell membrane, resulting in a purer PC extract [30]. Finally, the use of both DW and tap water resulted in very low purity ratios, likely due to the significant difference in osmotic pressure compared to the “Spirulina” culture medium. The relatively fragile cell wall of *Arthrospira* species, compared to other species, is probably responsible for the satisfactory yields of the extracted pigment [28]. Rolling two-way ANOVA (with a significance level of 0.05; Figure S1a) for all *A. platensis* scenarios revealed that the selected pH of the buffers had no significant effect on the extraction of the pigment after the first FT cycle, whereas the process was strongly affected by the type of extraction medium. 

### 2.2. Yield and Purity of Phycocyanin Extracted from Phormidium sp.

**Figure 2:**
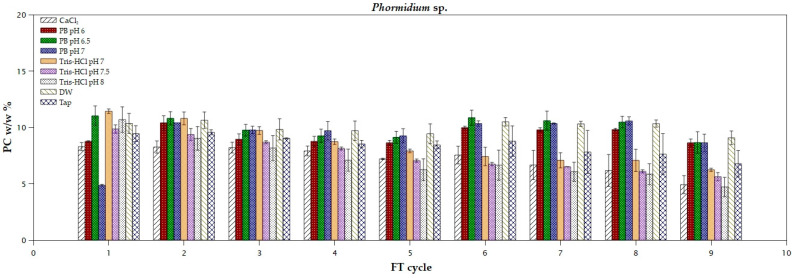
Extraction yields of phycocyanin from *Phormidium* sp. using different solvents during freeze and thaw cycles.

The marine cyanobacterium *Phormidium* sp. is a promising photosynthetic microorganism for PC production. As shown in Figure 2, most of the tested solutions can be used to extract the maximum pigment yield from the first FT cycle. More specifically, Tris-HCl pH 7, Tris-HCl pH 7.5, Tris-HCl pH 8, PB pH 6.5, DW, and tap water (*p* = 0.232) can be used to extract the maximum yield of PC equal to 10.5 ± 0.9% *w*/*w*. In the study of Mastropetros et al. [31], the percentage of the extracted PC, using deionized water and after the incubation of the lyophilized biomass of the same microorganism in the refrigerator for 24 h, was 0.9% *w*/*w*, indicating that the freeze–thaw method can significantly increase the extraction yield. The rolling two-way ANOVA of the results (Figure S1b) revealed no statistically significant difference (*p* > 0.05), using different solvents for the second until the fourth FT cycle. In another study, the maximum PC content from *Phormidium* sp. using 0.1 M phosphate buffer pH 7.1 after one FT cycle was 2.3% *w*/*w* [32]. Similar results, of 2–5% *w*/*w* PC, were also reported in other studies using phosphate buffers and the FT method, and a purity level of 0.55–0.69 [33,34]. Concerning the purity, CaCl_2_ exhibited the highest values and remained above 1 in all FT cycles, probably due to the same reasons as discussed in the case of *Arthrospira platensis* (Table 2). Nair et al. [35] achieved a purity ratio of 1.3 from *Phormidium valderianun* with the addition of activated charcoal and a PVDF membrane (0.45 μm). Corresponding to the case of *A. platensis*, a decrease in purity after the first FT cycle, using Tris-HCl buffer, could also be observed visually in *Phormidium* sp. from the color shift of the supernatant, from blue to light green. In general, the high extraction yield of PC from *Phormidium* sp. during the first cycle, even with tap water, that is reported in the present study, the need for sea water for its cultivation, and the easy harvesting of the biomass due to self-sedimentation, highlights the specific photosynthetic microorganism as a promising candidate for large-scale application.

### 2.3. Yield and Purity of Phycocyanin Extracted from Chlorogloeopsis fritschii

**Figure 3:**
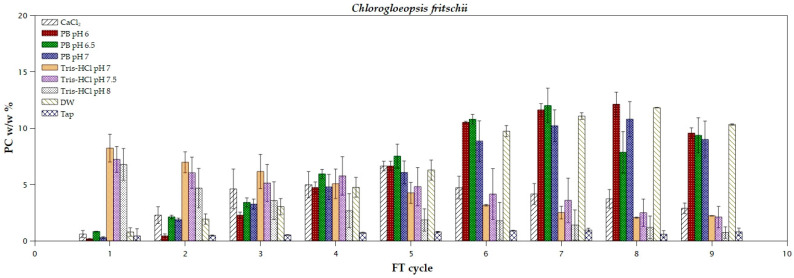
Extraction yields of phycocyanin from *Chlorogloeopsis fritschii* using different solvents during freeze and thaw cycles.

A two-way ANOVA was conducted on all *C. fritschii* scenarios, indicating that the pH values of the buffers did not have a significant impact on phycocyanin pigment extraction (Figure S1c). However, the type of extraction solution had a strong influence on the extracted pigment concentration. Among the tested solutions, Tris-HCl proved the most efficient for phycocyanin extraction from *Chlorogloeopsis fritschii*, as it yielded a sufficient amount (8.22% *w*/*w*, 0.8 purity) of the blue-colored pigment only from the very first cycle (Figure 3). As the FT cycles progressed, both concentration and purity started to drop (Figure 3; Table 3), possibly due to protein denaturation [17]. Previous research by Chittapun et al. utilized Tris-HCl buffer for phycocyanin extraction from cyanobacterium *Oscillatoria okeni* TISTR8549, achieving a 4% *w*/*w* yield after 18 FT cycles [13]. Nonetheless, phosphate buffer and DW yielded the highest phycocyanin concentration (11–12.8% *w*/*w*), but during the seventh FT cycle. Furthermore, both phosphate buffer and DW extraction, after five FT cycles, resulted in the highest pigment purities (0.9–1.04). Following a similar approach, Schipper et al. achieved, with Milli-Q^®^ water after four FT cycles, a phycocyanin yield of around 8% from cyanobacterium *Leptolyngbya* sp. [16]. To the best of our knowledge, only two studies have investigated extracting phycocyanin from *C. fritschii*. Silkina et al. achieved a concentration of around 9 μg mL^−1^ [36], and Candelo et al. obtained 0.87 mg mL^−1^ of phycocyanin using a cell disruptor and filtration [37].

### 2.4. Yield and Purity of Phycocyanin Extracted from Synechocystis sp.

**Figure 4:**
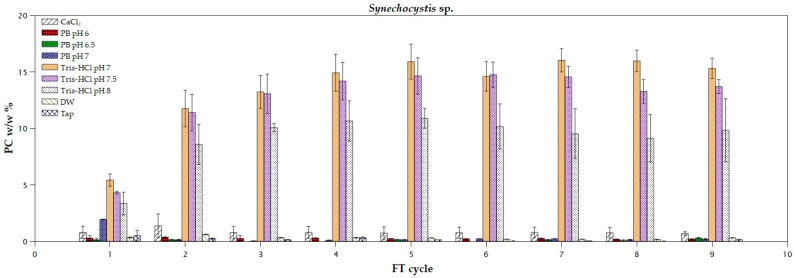
Extraction yields of phycocyanin from *Synechocystis* sp. using different solvents during freeze and thaw cycles.

In the case of *Synechocystis* sp., only Tris-HCl solution seemed to be effective for the extraction of PC (Figure 4). According to the rolling two-way ANOVA, both the used solvent and the pH seemed to affect the extraction yield (*p* < 0.05 for all the FT cycles; Figure S1d). Specifically for Tris-HCl, the pH of the solution did not affect the recovery of the product and could be considered statistically as one solvent. The highest extraction yield, 12.7 ± 2.4% *w*/*w*, was already achieved from the second FT cycle, as it was not significantly different from the following ones (*p* = 0.53). It is worth mentioning that a significant increase in extraction efficiency, equal to 147%, was achieved compared to the first cycle. Similar results were obtained by Imbibo et al., who reported a 14% *w*/*w* PC extraction from wet biomass of *Synechocystis* sp. PCC 6803 by using 50 mM sodium acetate buffer (pH 5.5) and applying ultrasonication for cell disruption [38]. In the study of Avci et al. [33], extraction of PC from lyophilized biomass of *Synechocystis* sp., using 0.01 M phosphate buffer (pH 7) and the FT method, was 0.4%, while when the bead-beating pretreatment method was applied, the extraction yield reached up to 17.5%. However, the applied pretreatment method strongly affects the extraction yield and depends on the morphology of the studied species. Focused pulsed treatment for cell disruption of *Synechocystis* sp. over 50 °C led to the denaturation of phycocyanin [39]. The highest purity of the pigment using Tris-HCl pH 7 and Tris-HCl pH 7.5 was obtained during the third cycle, equal to 1.2 and 1.0, respectively (Table 4). These values (>0.7) indicate that the extracted pigment meets the criteria for its direct use in food and cosmetic industries [40,41]. Purification methods, such as ultrafiltration, ammonium sulfate fractionation, and chromatography steps, can enhance the purity by removing the impurities that may be present in the extract [8]. Tris-HCl pH 8 did not affect the purity throughout the FT cycles. The maximum purity, reported in the literature, of the extracted PC from *Synechocystis* sp., obtained by using 0.1 M phosphate buffer (pH 7) after one FT cycle, was 0.7 [42]. In the case of *N. commune*, the purity ratio of PC, using 1 M Tris-HCl buffer (pH 8), decreased during all the FT cycles, while the opposite occurred for *O. okeni* [13]. 

In general, apart from the solvent used and the number of FT cycles, there are several physical and chemical parameters that affect the extraction efficiency of the process. Factors such as temperature, biomass/solvent ratio, biomass concentration, and its form also need to be optimized for a scale-up application [9]. Temperature and pH play an important role in the extraction efficiency, as they directly affect the protein. PC has an optimal extraction temperature of around 4 °C [43]. Higher temperatures, especially above 40 °C [43], can negatively impact the protein stability by disrupting its secondary, tertiary, and quaternary structure [9]. Moreover, while higher availability of the solvent can lead to higher extraction efficiencies, it can also impact the purity value due to the co-extraction of other pigments [18]. The form of biomass (dried or wet) is also crucial. Even though several studies have focused on PC extraction from dried biomass, drying methods can lead to a 50% loss of the pigment and have additional operational costs when scalable. Table 5 summarizes phycocyanin yield, purity, and extraction parameters from different microorganisms. 

## 3. Materials and Methods

Four cyanobacterial species from diverse habitats were investigated in this study: *Chlorogloeopsis fritschii* 1411-1a and *Arthrospira platensis* 21.99, acquired from the SAG Culture Collection (University of Göttingen), *Synechocystis* sp. PCC 6803, acquired from Pasteur Culture Collection of Cyanobacteria, and a local isolate of *Phormidium* sp., from Messolonghi, Western Greece [32]. Pure cultures of each cyanobacterium were preserved in 250 mL Erlenmeyer flasks (125 mL culture volume), capped with hydrophobic cotton, under constant illumination of 50 μmol photons m^−2^ s^−1^. BG-11 medium (73816, Sigma-Aldrich, St. Louis, MO, USA) enriched with trace element solution (Mix A5 with Co 92949, Sigma-Aldrich, USA) was used for *Chlorogloeopsis fritschii*, *Synechocystis* sp., and *Phormidium* sp. “Spirulina” medium was used for *Arthrospira platensis*, containing 13.61 g L^−1^ NaHCO_3_, 4.03 g L^−1^ Na_2_CO_3_, 0.5 g L^−1^ K_2_HPO_4_, 2.5 g L^−1^ NaNO_3_, 1 g L^−1^ K_2_SO_4_, 1 g L^−1^ NaCl, 0.2 g L^−1^ MgSO_4_ · 7H_2_O, 0.04 g L^−1^ CaCl_2_ · 2H_2_O, 0.01 g L^−1^ FeSO_4_ · 7H_2_O, 0.08 g L^−1^ EDTA, and 0.5% *v*/*v* micronutrient solution [48]. Deionized water was used to prepare cyanobacterial mediums, except for *Phormidium* sp., where autoclaved and filtered (with Whatman GF/F, pore size 0.7 µm) seawater (37 ppt salinity) was used, collected from Kastellokampos Beach, Patras, Greece.

To obtain cyanobacterial biomass for each extraction scenario, 400 mL of autotrophic culture was prepared in 500 mL Erlenmeyer flasks for each species, with an initial cell density of 0.2 g L^−1^. Filtered atmospheric air (PTFE filter, 0.22 μm) was provided to the cultures at a mass flow rate of 0.5 L min^−1^. The temperature was kept at 30 °C, and a 16:8 light/dark cycle was applied using cold white LED bulbs with a photosynthetic photon flux density of 100 μmol photons m^−2^ s^−1^. Once the cultures achieved the desired biomass concentration (1.1 ± 0.2 g L^−1^), after about 9 days from inoculation, the cells were harvested through centrifugation for 5 min at 4500 rpm (Hermle Z 366, Hermle Labortechnik GmbH, Wehingen, Germany) and the supernatant was discarded. For washing, deionized water was used for *C. fritschii* and *Synechocystis* sp., while a 0.5 M ammonium bicarbonate (NH_4_HCO_3_) solution was used for *Phormidium* sp. and *A. platensis*. After washing, the harvested biomass was resuspended in different extraction solutions. Total suspended solids (TSS) were calculated according to Standard Methods [49], in order to determine the biomass concentration (g L^−1^) by weighting a wet sample amount of the culture before and after 105 °C, until constant weight was achieved, using Whatman Glass Microfiber Filters (0.7 μm pore size). 

**Figure 5:**
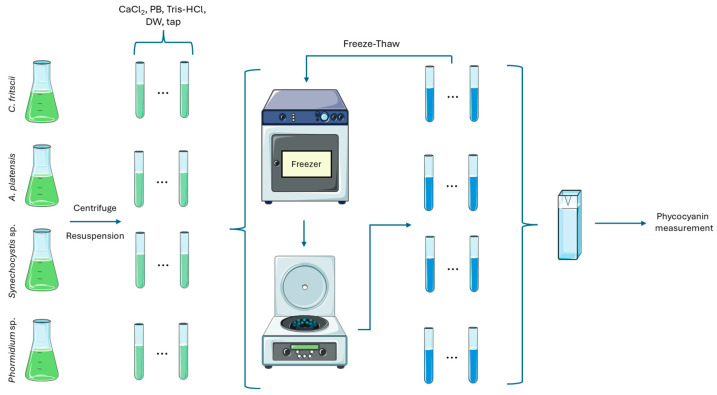
Schematic representation of the applied methodology.

To evaluate the extraction of phycocyanin from the cyanobacterial biomass (Figure 5), seven distinct aquatic solutions were prepared: (1) 0.1 M CaCl_2_, (2) 0.5 M sodium phosphate buffer (PB) pH 6, (3) 0.5 M sodium phosphate buffer pH 6.5, (4) 0.5 M sodium phosphate buffer pH 7, (5) 1 M Tris-HCl buffer pH 7, (6) 1 M Tris-HCl buffer pH 7.5, and (7) 1 M Tris-HCl buffer pH 8. The pH values of the buffer solutions were chosen based on the optimal range for protein stability (pH 6–8) [9]. Additionally, deionized (DW) and tap water were also used as extraction media. A 1:1 culture-to-solvent ratio was chosen and, therefore, 4 mL of each culture, after being centrifuged and washed, was finally resuspended in 4 mL of extraction solution. The suspension was vortexed and stored in the freezer (−20 °C). Following freezing, the samples were thawed at 4 °C, over a 24 h period in the dark. This freeze–thaw cycle was repeated a total of 10 times, utilizing the same supernatant extraction solution for the phycocyanin (PC) measurement of each cycle.

The absorbance of the supernatant was measured with a UV-VIS spectrophotometer (Cary50, Varian, Palo Alto, CA, USA) at 4 different wavelengths of 280 nm, 620 nm, 652 nm, and 750 nm in order to determine the phycocyanin content. The PC concentration was quantified using the following equation [50]:

(1) $$PC\;[mg\cdot {mL}^{-1}]=\frac{A_{620nm}-0.474\cdot A_{652nm}}{5.34}$$

The yield of the extracted pigment was expressed as:

(2) $$PC[w/w\%]=\frac{PC\;[mg\cdot {mL}^{-1}]}{TSS\;[mg\cdot {mL}^{-1}]}\cdot 100\%$$

The purity of the extracted pigment was calculated as the ratio of A_620nm_ to A_280nm_, as the proteins absorb at 280 nm due to aromatic amino acids [50,51]. The absorption at 750 nm was used for background corrections. The applied methodology is presented in Figure 5.

All experiments were performed in duplicate, and the results are presented as average values along with the calculated standard deviation. Experimental data for phycocyanin recovery were examined for their statistical differences through a rolling two-way ANOVA [52,53]. The analysis was performed in terms of different solvents used and pH levels and is presented in the Appendix. One-way ANOVA and Tukey’s test were also performed to examine the statistical difference between the data concerning phycocyanin purity. In both cases, significance was established at *p* < 0.05 (null hypothesis). The analysis was performed via Minitab 18 software. 

## 4. Conclusions

In the present study, the blue pigment phycocyanin was successfully extracted from all four cyanobacterial species using the freeze and thaw method in different extraction solutions. The freeze and thaw method proved to be a simple and effective method for disrupting the cell walls of cyanobacteria from various environments, facilitating the extraction of phycocyanin. The optimal extraction solvent and freeze–thaw cycles for cyanobacteria appeared to be highly species-dependent. During the present study, the following highest yields were achieved: 11.3% *w*/*w* from *Arthrospira platensis*, 10.5% *w*/*w* from marine cyanobacterium *Phormidium* sp., 11.8% from *Chlorogloeopsis fritschii*, and 16.0% from *Synechocystis* sp. Tris-HCl buffer appeared to be the most suitable for a rapid extraction of phycocyanin across all species, while in the case of *Phormidium* sp., the maximum recovery yield could be achieved even with tap water from the first cycle, enhancing the economic feasibility of the process. However, CaCl_2_ exhibited high purity ratios, even in cases where the maximum yield was not achieved. In all cases, purity ratios indicated that the extracted phycocyanin could be characterized as food grade (>0.7) but not as analytical grade (>4.0).

This study provided valuable insights into phycocyanin extraction from cyanobacteria by demonstrating the effectiveness of the simple freeze–thaw method. A techno-economic analysis considering the used solvent, number of freeze–thaw cycles, and extraction efficiency combined with the purity of the extracted pigment would be valuable in decision-making for selecting the optimal phycocyanin extraction process for each cyanobacterial species. Moreover, the results of this study suggested that probably, the differences in cell morphology across species complicated the application of the exact same methodology for PC extraction in all species. Genetic modification of species might provide a uniform extraction process, facilitating large-scale application. Further research on cultivating cyanobacteria in wastewater for phycocyanin production can be a promising solution to enhancing the economic feasibility of the process.

Phycocyanin extraction is just one step in its production. Further research is crucial for both upstream and downstream processes. This includes optimizing cyanobacteria culture parameters and improving extraction and purification methods. Additionally, developing and implementing a standardized analytical protocol for quantitative and qualitative analysis of phycocyanin is essential for environmental, industrial, and research applications.

## Figures and Tables

**Table 1:** Purity ratios for *Arhtrospira platensis* phycocyanin extract.

Cycle	1st	2nd	3rd	4th	5th	6th	7th	8th	9th
Solution									
**CaCl_2_**	0.92 ± 0.14 ^a^	0.66 ± 0.07 ^a,b^	0.71 ± 0.11 ^a,b^	0.69 ± 0.01 ^a,b^	0.61 ± 0.01 ^a,b^	0.58 ± 0.09 ^a,b^	0.54 ± 0.09 ^b^	0.51 ± 0.08 ^b^	0.46 ± 0.08 ^b^
**PB pH 6**	0.27 ± 0.10 ^a^	0.78 ± 0.09 ^a^	0.77 ± 0.11^a^	0.74 ± 0.15 ^a^	0.75 ± 0.16 ^a^	0.70 ± 0.16 ^a^	0.67 ± 0.14 ^a^	0.65 ± 0.12 ^a^	0.64 ± 0.13 ^a^
**PB pH 6.5**	0.67 ± 0.21 ^a^	0.72 ± 0.24 ^a^	0.73 ± 0.22 ^a^	0.61 ± 0.12 ^a^	0.61 ± 0.13 ^a^	0.59 ± 0.11 ^a^	0.59 ± 0.11 ^a^	0.59 ± 0.12 ^a^	0.56 ± 0.13 ^a^
**PB pH 7**	0.30 ± 0.07 ^a^	0.71 ± 0.15 ^a^	0.64 ± 0.14 ^a^	0.60 ± 0.13 ^a^	0.59 ± 0.13 ^a^	0.54 ± 0.12 ^a^	0.54 ± 0.13 ^a^	0.52 ± 0.11 ^a^	0.50 ± 0.13 ^a^
**Tris-HCl pH 7**	0.62 ± 0.09 ^a^	0.47 ± 0.08 ^a,b^	0.45 ± 0.07 ^a,b^	0.44 ± 0.08 ^a,b^	0.42 ± 0.08 ^a,b^	0.41 ± 0.08 ^a,b^	0.36 ± 0.07 ^a,b^	0.34 ± 0.06 ^a,b^	0.31 ± 0.05 ^b^
**Tris-HCl pH 7.5**	0.57 ± 0.12 ^a^	0.44 ± 0.06 ^a,b^	0.41 ± 0.06 ^a,b^	0.37 ± 0.07 ^a,b^	0.35 ± 0.06 ^a,b^	0.33 ± 0.06 ^a,b^	0.29 ± 0.05 ^b^	0.28 ± 0.05 ^b^	0.25 ± 0.04 ^b^
**Tris-HCl pH 8**	0.45 ± 0.03 ^a^	0.38 ± 0.07 ^a,b^	0.34 ± 0.06 ^a,b,c^	0.28 ± 0.05 ^b,c,d^	0.24 ± 0.03 ^b,c,d^	0.22 ± 0.02 ^b,c,d^	0.19 ± 0.01 ^c,d^	0.17 ± 0.01 ^d^	0.16 ± 0.01 ^d^
**DW**	0.22 ± 0.08 ^a^	0.15 ± 0.02 ^a^	0.13 ± 0.02 ^a^	0.12 ± 0.02 ^a^	0.11 ± 0.02 ^a^	0.11 ± 0.01 ^a^	0.11 ± 0.01 ^a^	0.10 ± 0.01 ^a^	0.10 ± 0.01 ^a^
**Tap Water**	0.41 ± 0.03 ^a^	0.27 ± 0.03 ^a,b^	0.22 ± 0.02 ^b^	0.17 ± 0.00 ^b^	0.13 ± 0.00 ^b^	0.12 ± 0.01 ^b^	0.09 ± 0.01 ^b^	0.09 ± 0.01 ^b^	0.08 ± 0.01 ^b^

^a,b,c,d^ Data in the same row that share a common lowercase letter are not significantly different (Tukey Test).

**Table 2:** Purity ratios for *Phormidium* sp. phycocyanin extract.

Cycle	1st	2nd	3rd	4th	5th	6th	7th	8th	9th
Solution									
**CaCl_2_**	1.50 ± 0.07 ^a^	1.39 ± 0.10 ^a^	1.46 ± 0.09 ^a^	1.45 ± 0.16 ^a^	1.46 ± 0.25 ^a^	1.42 ± 0.23 ^a^	1.30 ± 0.23 ^a^	1.21 ± 0.28 ^a^	1.07 ± 0.31 ^a^
**PB pH 6**	0.73 ± 0.01 ^b,c^	0.57 ± 0.00 ^d^	0.75 ± 0.00 ^a,b^	0.74 ± 0.00 ^b,c^	0.77 ± 0.01 ^a^	0.75 ± 0.00 ^ab^	0.77 ± 0.00 ^a^	0.77 ± 0.01 ^a^	0.72 ± 0.01 ^c^
**PB pH 6.5**	0.61 ± 0.02 ^b^	0.52 ± 0.00 ^c^	0.67 ± 0.00 ^a,b^	0.67 ± 0.02 ^a,b^	0.71 ± 0.02 ^a^	0.68 ± 0.02 ^a,b^	0.67 ± 0.03 ^a,b^	0.67 ± 0.02 ^a,b^	0.66 ± 0.00 ^a,b^
**PB pH 7**	0.47 ± 0.03 ^c^	0.55 ± 0.05 ^b,c^	0.61 ± 0.04 ^a,b^	0.61 ± 0.00 ^a,b^	0.67 ± 0.02 ^a,b^	0.68 ± 0.03 ^a^	0.67 ± 0.05 ^a,b^	0.61 ± 0.02 ^a,b^	0.60 ± 0.03 ^a,b,c^
**Tris-HCl pH 7**	0.50 ± 0.01 ^a^	0.43 ± 0.00 ^b^	0.41 ± 0.01 ^c^	0.39 ± 0.01 ^c^	0.37 ± 0.00 ^d^	0.36 ± 0.00 ^d^	0.31 ± 0.00 ^e^	0.29 ± 0.00 ^f^	0.26 ± 0.00 ^g^
**Tris-HCl pH 7.5**	0.49 ± 0.01 ^a^	0.44 ± 0.02 ^b^	0.40 ± 0.00 ^c^	0.38 ± 0.01 ^c,d^	0.36 ± 0.00 ^d,e^	0.33 ± 0.00 ^e^	0.30 ± 0.00 ^f^	0.30 ± 0.00 ^f^	0.26 ± 0.00 ^g^
**Tris-HCl pH 8**	0.49 ± 0.03 ^a^	0.39 ± 0.03 ^b^	0.37 ± 0.03 ^b,c^	0.34 ± 0.02 ^b,c,d^	0.32 ± 0.03 ^b,c,d,e^	0.30 ± 0.02 ^c,d,e,f^	0.26 ± 0.01 ^d,e,f^	0.24 ± 0.00 ^e,f^	0.23 ± 0.00 ^f^
**DW**	0.68 ± 0.04 ^a,b^	0.59 ± 0.01 ^b^	0.70 ± 0.03 ^a,b^	0.69 ± 0.03 ^a,b^	0.72 ± 0.04 ^a^	0.73 ± 0.03 ^a^	0.71 ± 0.00 ^a^	0.67 ± 0.03 ^ab^	0.66 ± 0.01 ^a,b^
**Tap Water**	0.77 ± 0.27 ^a^	0.59 ± 0.19 ^a^	0.65 ± 0.22 ^a^	0.66 ± 0.21 ^a^	0.67 ± 0.22 ^a^	0.64 ± 0.21 ^a^	0.67 ± 0.23 ^a^	0.61 ± 0.28 ^a^	0.59 ± 0.31 ^a^

^a,b,c,d,e,f^ Data in the same row that share a common lowercase letter are not significantly different (Tukey Test).

**Table 3:** Purity ratios for *Chlorogloeopsis fritschii* phycocyanin extract.

Cycle	1st	2nd	3rd	4th	5th	6th	7th	8th	9th
Solution									
**CaCl_2_**	0.17 ± 0.09 ^b^	0.50 ± 0.12 ^ab^	0.80 ± 0.23 ^a^	0.79 ± 0.12 ^a^	0.72 ± 0.07 ^a^	0.65 ± 0.05 ^a^	0.57 ± 0.04 ^ab^	0.48 ± 0.04 ^ab^	0.39 ± 0.04 ^ab^
**PB pH 6**	0.02 ± 0.01 ^d^	0.08 ± 0.02 ^c,d^	0.44 ± 0.02 ^b,c^	0.76 ± 0.04 ^a,b^	0.96 ± 0.11 ^a^	1.02 ± 0.09 ^a^	1.03 ± 0.13 ^a^	1.04 ± 0.15 ^a^	0.99 ± 0.15 ^a^
**PB pH 6.5**	0.22 ± 0.11 ^c^	0.40 ± 0.21 ^b,c^	0.71 ± 0.11 ^a,b,c^	0.90 ± 0.04 ^a,b^	1.01 ± 0.06 ^a^	1.04 ± 0.14 ^a^	1.00 ± 0.14 ^a^	0.78 ± 0.14 ^a,b^	0.70 ± 0.14 ^a,b,c^
**PB pH 7**	0.10 ± 0.01 ^b^	0.39 ± 0.35 ^a,b^	0.60 ± 0.33 ^a,b^	0.79 ± 0.26 ^a,b^	0.88 ± 0.22 ^a,b^	0.94 ± 0.15 ^a,b^	0.94 ± 0.14 ^a^	0.95 ± 0.11 ^a^	0.92 ± 0.10 ^a,b^
**Tris-HCl pH 7**	0.80 ± 0.14 ^a^	0.59 ± 0.14 ^a,b^	0.50 ± 0.14 ^a,b^	0.39 ± 0.15 ^a,b^	0.32 ± 0.14 ^a,b^	0.25 ± 0.14 ^b^	0.20 ± 0.13 ^b^	0.16 ± 0.12 ^b^	0.13 ± 0.10 ^b^
**Tris-HCl pH 7.5**	0.67 ± 0.14 ^a^	0.47 ± 0.15 ^a,b^	0.41 ± 0.16 ^a,b^	0.31 ± 0.16 ^a,b^	0.25 ± 0.15 ^a,b^	0.19 ± 0.13 ^a,b^	0.15 ± 0.10 ^b^	0.10 ± 0.04 ^b^	0.10 ± 0.07 ^b^
**Tris-HCl pH 8**	0.64 ± 0.14 ^a^	0.38 ± 0.16 ^a,b^	0.30 ± 0.15 ^a,b^	0.22 ± 0.13 ^a,b^	0.14 ± 0.06 ^a,b^	0.14 ± 0.09 ^a,b^	0.11 ± 0.07 ^b^	0.09 ± 0.05 ^b^	0.05 ± 0.03 ^b^
**DW**	0.21 ± 0.15 ^d^	0.37 ± 0.00 ^c,d^	0.61 ± 0.01 ^b,c^	0.76 ± 0.01 ^a,b^	0.91 ± 0.04 ^a,b^	0.97 ± 0.06 ^a^	1.02 ± 0.09 ^a^	1.01 ± 0.10 ^a^	0.97 ± 0.09 ^a^

^a,b,c,d^ Data in the same row that share a common lowercase letter are not significantly different (Tukey Test).

**Table 4:** Phycocyanin purities for *Synechocystis* sp. phycocyanin extract.

Cycle	1st	2nd	3rd	4th	5th	6th	7th	8th	9th
Solution									
**Tris-HCl pH 7**	0.61 ± 0.02 ^d^	1.04 ± 0.04 ^c^	1.21 ± 0.07 ^a,b^	1.33 ± 0.02 ^a^	1.37 ± 0.02 ^a^	1.29 ± 0.01 ^a^	1.29 ± 0.01 ^a^	1.18 ± 0.05 ^a^	1.08 ± 0.08 ^c^
**Tris-HCl pH 7.5**	0.49 ± 0.02 ^d^	0.93 ± 0.08 ^b,c^	1.09 ± 0.09 ^a,b^	1.14 ± 0.08 ^a,b^	1.17 ± 0.08 ^a^	1.13 ± 0.02 ^a,b^	1.02 ± 0.04 ^a,b,c^	1.00 ± 0.05 ^a,b,c^	0.82 ± 0.03 ^c^
**Tris-HCl pH 8**	0.39 ± 0.02 ^a^	0.57 ± 0.20 ^a^	0.68 ± 0.25 ^a^	0.74 ± 0.24 ^a^	0.81 ± 0.18 ^a^	0.75 ± 0.01 ^a^	0.73 ± 0.16 ^a^	0.69 ± 0.15 ^a^	0.60 ± 0.18 ^a^

^a,b,c,d^ Data in the same row that share a common lowercase letter are not significantly different (Tukey Test).

**Table 5:** Phycocyanin yield, purity, and extraction parameters from different microorganisms.

Microorganism	Extraction Solvent	Freeze–Thaw Cycles	Phycocyanin Yield	Purity	Reference
*Arthrospira (Spirulina) platensis*	Sodium Phosphate buffer 0.1 M pH 7	3	146 mg g^−1^	0.8	[44]
*Arthrospira (Spirulina)* sp. LEB18	Tris-HCl buffer 10 mM pH 8.3	2	58 mg g^−1^	n.a.	[45]
		4	101 mg g^−1^		
*Arthrospira platensis*	Sodium Phosphate buffer 50 mM pH 7 + 1 mM sodium azide	1	175 mg g^−1^	0.8	[34]
*Arthrospira platensis* SAG 21.99	Tris-HCl buffer 1 M pH 7	1	113 mg g^−1^	0.6	This study
*N. commune* TUBT05	Tris-HCl buffer 1 M pH 8	3	27.4 mg g^−1^	0.5	[13]
	Sodium Phosphate buffer 0.1 M pH 7	3	29.7 mg g^−1^	0.6	
*Chlorogloeopsis fritschii* SAG 1411–1a	Tris-HCl buffer 1 M pH 7	1	82.2 mg g^−1^	0.8	This study
	Sodium Phosphate buffer 0.1 M pH 6.5	7	120 mg g^−1^	1	
*Leptolyngbya* sp. QUCCCM 56	Milli-Q water	4	80.1 mg g^−1^	2	[16]
*Porphyridium* sp.	Modified F2 medium	1	0.1 mg mL^−1^	n.a.	[46]
*Euhalothece* sp.	Sodium Phosphate buffer 50 mM pH 7	1	35.3 mg g^−1^	>2	[47]
	Phosphate Buffer Saline	1	31.1 mg g^−1^	n.a.	
*Synechocystis* sp. PCC 6803	Tris-HCl buffer 1 M pH 7	2	117 mg g^−1^	1	This study
*Phormidium* sp.	Sodium Phosphate buffer 0.5 M pH 7	1	22.6 mg g^−1^	n.a.	[32]
*Phormidium* sp.	Sodium Phosphate buffer 50 mM pH 7 1 mM sodium azide	1	41 mg g^−1^	0.7	[34]
*Phormidium* sp.	Potassium Phosphate buffer 10 mM pH 7	1	48 mg g^−1^	0.6	[33]
*Phormidium* sp.	0.1 M CaCl_2_	1	83 mg g^−1^	1.5	This study
	Deionized water	1	104 mg g^−1^	0.7	

n.a.: Not available.

## Data Availability

Data will be made available upon request.

## Supplementary Materials

The following supporting information can be downloaded at: https://www.mdpi.com/article/10.3390/md22060246/s1, Figure S1. Rolling two-way ANOVA (*p*-values) for different solvents and pH for: (**a**) *Arthrospira platensis*, (**b**) *Phormidium* sp., (**c**) *Chlorogloeopsis fritschii*, and (**d**) *Synechocystis* sp.
