# Supplementary material for: Optimizing Phycocyanin Extraction from Cyanobacterial Biomass: A Comparative Study of Freeze–Thaw Cycling with Various Solvents
Source: Mar Drugs. 2024 May 28;22(6):246. doi: 10.3390/md22060246 (PMC11204620; doi:10.3390/md22060246)
Supplement: Supplementary file 1 [file marinedrugs-22-00246-s001.zip › marinedrugs-3014292-supplementary.pdf]

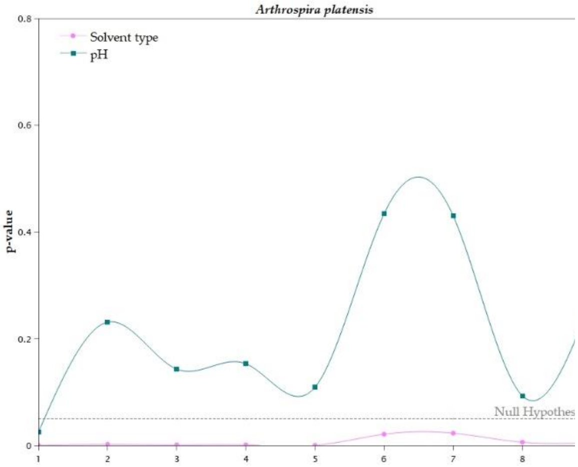


(**a**)

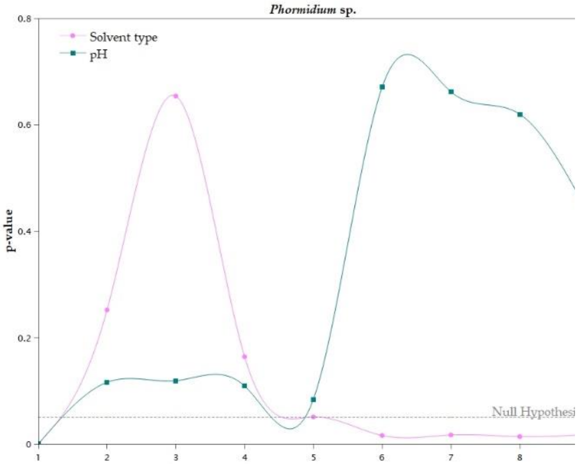


(**b**)

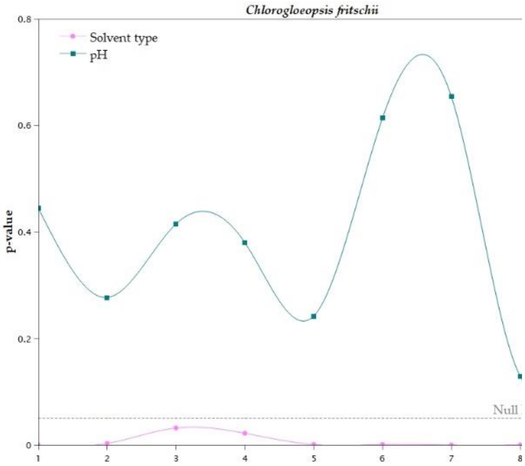


(**c**)

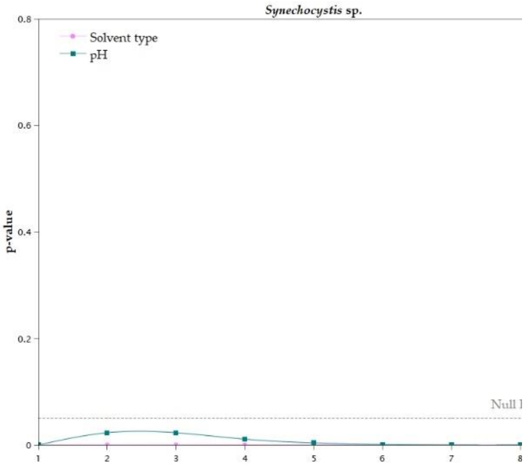


(**d**)

**Figure S1.** Rolling two-way ANOVA (*p*-values) for different solvents and pH for: (**a**) *Arthrospira platensis*, (**b**) *Phormidium* sp., (**c**) *Chlorogloeopsis fritschii*, and (**d**) *Synechocystis* sp.
